# Repeatability and Comparability of Retinal Blood Vessel Caliber Measurements by OCTA

**DOI:** 10.3390/vision7030048

**Published:** 2023-07-03

**Authors:** Joby Tsai, Samuel Asanad, Martha Whiting, Xuemin Zhang, Laurence Magder, Osamah Saeedi

**Affiliations:** 1 Department of Ophthalmology, Broward Health, Deerfield Beach, FL 33064, USA; 2 Department of Ophthalmology and Visual Sciences, University of Maryland, Baltimore, MD 21201, USA; 3 Department of Epidemiology and Public Health, University of Maryland School of Medicine, Baltimore, MD 21201, USA

**Keywords:** optical coherence tomography angiography (OCTA), primary open-angle glaucoma (POAG), repeatability, vessel caliber

## Abstract

Background: To investigate the repeatability in vessel caliber measurements by optical coherence tomography angiography (OCTA). Methods: In this prospective study, 28 patients (47 eyes) underwent sequential OCTA imaging of the optic nerve head and macula. Two independent masked graders measured vessel caliber for sequential images of the optic nerve head and macula. The average vessel width was determined and variability between graders and images. Results: A total of 8400 measurements of 420 vessels from 84 OCTA images were included in the analysis. Overall, inter-grader agreement was excellent (ICC 0.90). The coefficient of variation (CoV) for all repeated OCTA images was 0.10. Greater glaucoma severity, older age, macular location, and diagnosis of diabetes were associated with thinner vessels (*p* < 0.05). CoV was higher in the peripapillary region (0.07) as compared to the macula (0.15). ICC was high for all subgroups except for the macula (ICC = 0.72). Conclusions: Overall, the repeatability of vessel caliber measurements by OCTA was high and variability low. There was greater variability in the measurement of macular vessels, possibly due to technical limitations in acquiring accurate vessel widths for smaller macular vessels.

## 1. Introduction

Optical coherence tomography angiography (OCTA) is a noninvasive imaging technology that allows visualization of the retinal vasculature down to the capillary level at varying retinal and choroidal depths [1]. There is a critical need for establishing precision in imaging of the retinal vasculature with OCTA, as various quantifiable metrics have been shown to have important practical implications for earlier detection of disease [2,3]. Among these, vessel density (VD) and perfusion density (PD) are two surrogate markers for retinal perfusion, which can be measured with OCTA using a selected pixel threshold [4]. Previous OCTA studies have shown reduced VD and PD in various ocular pathologies including age-related macular degeneration (ARMD) [5], diabetic retinopathy [6], and glaucoma [7,8]. Thereby suggesting a promising role for OCTA in the diagnosis and monitoring of disease progression [9].

In addition to VD and PD, retinal vessel caliber is another potentially important for diagnosing and even monitoring the treatment of ophthalmologic diseases. Recent studies have suggested the clinical utility of vessel caliber in various ocular and systemic conditions such as diabetic retinopathy [3,10], birdshot chorioretinopathy [11], and cardiovascular disease [3,12]. Retinal arteriolar narrowing is one of the earliest signs of hypertension and may be useful in predicting risk of cardiovascular disease [13]. Aside from its diagnostic and prognostic values, vessel caliber is also necessary for accurately estimating retinal blood flow [14,15]. Notably, previous studies have illustrated that retinal blood flow is impaired in glaucoma and that dysregulation of vascular flow in sick or damaged retinal ganglion cells may precede structural reduction from retinal ganglion cell (RGC) loss [16]. In turn, given the potential importance of vessel caliber as a biomarker, it is important to establish the reliability of OCTA for vessel caliber measurements.

Various imaging modalities have been used for measuring vessel caliber including infrared reflectance images [17], spectral domain (SD-OCT) [17,18], scanning laser ophthalmoscopy [19], fundus photography [10,11,12,13,15,20,21,22,23,24,25,26,27,28,29], and traditional angiography [30,31]. Relative to fundus photography, OCTA reports larger values for vessel caliber measurements [22]. These larger values may result from the limited resolution and width of the OCTA beam (14 microns) [32,33]. Variability in OCTA measurements may also arise from a combination of physiological and technical factors. Physiologically, vessel caliber varies with vessel pulsation as a function of the cardiac cycle [20]. Since OCTA image acquisition can last up to two minutes [32], which spans over multiple cardiac cycles, the scan produced represents an undefined time in systole, diastole, or somewhere in between [33]. Given these dynamic physiologic changes in vessel caliber, OCTA images may be susceptible to motion artifacts from the axial displacement of the retina during systole. Moreover, since image resolution is limited by the size of the illumination beam [33], smaller vessel calibers are affected proportionately more than larger vessel calibers [34]. Studies of these artifacts in aggregate on vessel caliber repeatability and variability as measured by OCTA are limited and remain unclear.

Given that OCTA is a relatively new imaging modality, there is still need for data on its repeatability [34] and reproducibility [34]. Preliminary studies have found excellent repeatability and reproducibility of two important OCTA metrics, VD and PD [34]. Though reassuring, there are various limitations to be mindful of when assessing ocular vascular pathology. VD calculates the percentage area occupied by vessels, which inherently depends on both vessel length and vessel caliber. Therefore, it is possible that VD would remain unchanged in a circumstance of decreased perfusion and simultaneous vessel dilation, resulting in a false negative finding of a potential vascular abnormality. [35] In addition, PD solely evaluates vessel length without considering vessel caliber [35]. In contrast, vessel diameter index (VDI) is derived using both VD and PD, and it has been shown as a sensitive and reproducible OCTA metric for vessel caliber [36]. However, VDI is similarly not without potential shortcomings as this index reflects vessel caliber averaged over an entire scan. It is possible that the individual vessels within a given scan can exhibit differences in vessel caliber such that some may constrict, while others may dilate, similarly resulting in a false negative finding overall. Since VDI provides an average of vessel caliber across many vessels, this index may not accurately reflect the repeatability of vessel caliber at the level of a single vessel. The purpose of the present article is to evaluate the repeatability and variability of OCTA vessel caliber using consecutively obtained OCTA images as an isolated metric at the level of single vessels in both the peripapillary and macular regions.

## 2. Materials and Methods

### 2.1. Subjects

We conducted a prospective, cross-sectional study of 28 patients (47 eyes) from clinics at the University of Maryland Department of Ophthalmology and Visual Sciences from November 2017 to January 2018. Informed consent was obtained for each subject and approval was obtained from the Institutional Review Board of the University of Maryland. This study adhered to the tenets of the Declaration of Helsinki and the Health Insurance Portability and Accountability Act. All subjects were older than 18 years, had no significant ocular media opacities, and had no problems with visual fixation. All subjects had an extensive ophthalmologic examination, including best-corrected visual acuity, intraocular pressure, Humphrey visual field testing, slit-lamp biomicroscopy, and ophthalmoscopy. Patients with significant ocular media opacities and fixation deficits precluding imaging were excluded from the study. Patient diagnoses were confirmed through chart review.

### 2.2. Imaging

OCTA imaging was performed using the Heidelberg Spectralis (Heidelberg Engineering, Heidelberg, Germany) in a dimly lit room. OCTA scans were acquired in a 10 × 10-degree field of view focused on the first optic nerve head and, subsequently, the fovea using a raster scan with 11-micron spacing averaged five times for the superficial vascular complex. For all subjects, two sequential scans in each location in each eye were acquired for during a single visit within two minutes of each other. The images were registered on acquisition to ensure that the exact same location was imaged.

### 2.3. Analysis

#### 2.3.1. ImageJ and Image Quality—Processing and Vessel Identification

**Figure 1:**
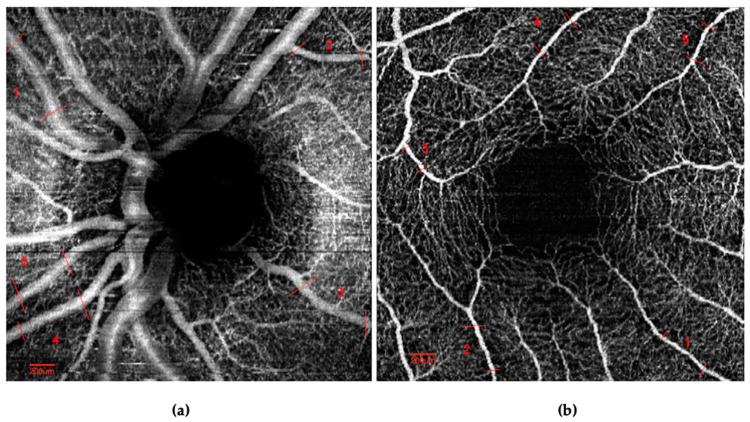
Sample healthy control subject OCTA image of (**a**) optic disc and (**b**) macula with 5 non-branching vessels labeled for caliber measurements.

ImageJ software (Version 1.51 NIH, Bethesda, MD 20892, USA) was used for vessel caliber analysis. Five vessel segments in the peripapillary and macular regions centered around the optic nerve head and the fovea, respectively, were pre-marked by two masked graders of the highest quality (JT, XZ) (Figure 1). We included only vessels that were well-defined and at least 400 µm in length and excluded branched, merged, or distorted vessels.

#### 2.3.2. Image Quality Grading

Each image pair was evaluated for image quality using a three-tier grading system (Poor, Fair, Good). Poor was defined as images having greater than 50% distortion or non-discernible vessel edges spanning continuously over 200 µm in length. Fair was defined as images having between 20 and 50% distortion and clear vessel edges at least 200 µm in length. Good was defined as images having no greater than 20% distortion and clear vessel edges that were continuous throughout. This would help to account for any motion artifact. Table 1 outlines the total number of images in each quality category. See Supplementary Materials Section (Figures S1–S3, Tables S1 and S2) for examples of Poor, Good, and Fair images.

Each image was checked for segmentation errors. Based on the image quality grading criteria, images that had breaks in vessels, unclear vessels, projection artifacts, or segmentation errors were not accepted. As this study specifically focuses on larger vessels in the superficial plexus, it is unlikely that a segmentation error would have a significant effect on measurements. All OCTAs had a quality rating greater than 25 and the average quality rating was 34.5.

The OCTA quality scores from the device represent the average signal-to-noise ratio of the OCTA volume. However, they do not account for possible focus errors, suboptimal optical alignment, and layer segmentation errors. Therefore, these quality scores were insufficient to determine the overall image quality and other factors that were outlined prior were also reasons for exclusion despite high quality scores.

#### 2.3.3. Vessel Caliber Measurements

**Figure 2:**
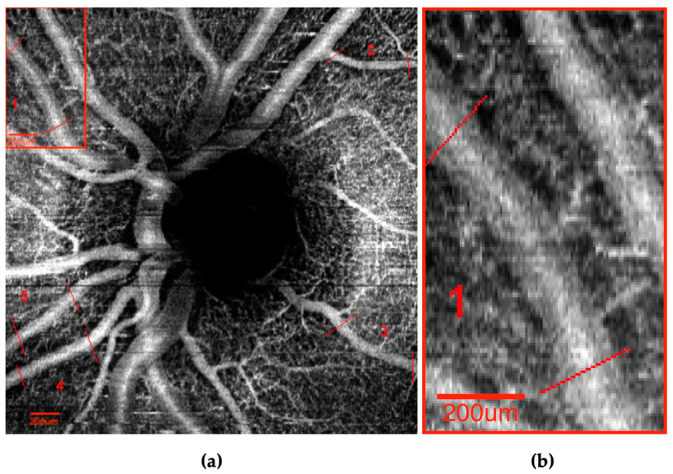
(**a**) Sample optic nerve head OCTA image. (**b**) Magnified view of vessel caliber measurements made for “vessel 1”.

**Figure 3:**
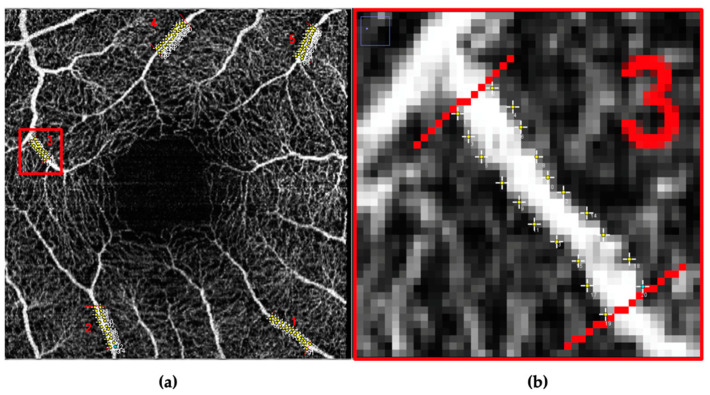
(**a**) Sample macula OCTA image. (**b**) Magnified view of vessel caliber measurements for “vessel 3”.

Marked OCTA image pairs were independently graded by two experienced, masked graders (JT, XZ), and both horizontal and vertical vessels were analyzed. To calculate the interclass correlation coefficient (ICC), approximately 50% of the images were graded by both graders. Vessel caliber was defined as the distance between vessel edges measured at five-pixel intervals along the length of the marked vessels for both optic nerve (Figure 2) and macula (Figure 3) images using the ImageJ point tool.

#### 2.3.4. Vessel Segment Length Measurements

**Figure 4:**
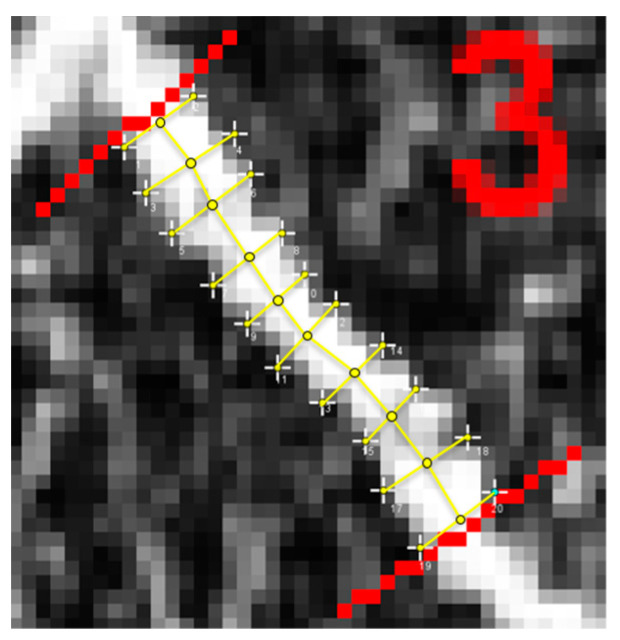
Measurement of vessel segment length for calculating the distance between the midpoints of the vessel caliber coordinate pairs.

As shown in Figure 4, vessel length was defined as the total distance measured from the center of a given vessel segment. This distance was determined by summing the individual vessel lengths measured at five-pixel increments. The lengths of these vessel caliber segments were measured by summing the distance between the midpoints of the individual caliber measurements (Figure 4). The midpoint coordinates of each vessel were defined as the average of the two X and Y coordinates. The distance between consecutive midpoint coordinates was then calculated.

### 2.4. Statistical Methods

For each vessel in each image and each grader, vessel caliber was calculated as the average of all the individual caliber measurements for that vessel. The standard deviation (SD) of two caliber measures of the same vessel by different graders was estimated using mixed effects models. These “measurement standard deviations” were then converted to ICCs or Coefficients of Variation (CoV). The ICC was defined as the variance of vessel widths divided by the sum of the variance of vessel width and the between-grader variance in vessel width measure (i.e., the square of the measurement SD). The sample size was based on available patients, staff, and time. The resulting sample size for estimating the ICC was quite large and resulted in good precision for our estimates. Specifically, the ICC was estimated based on 550 vessels each imaged twice. This results in an ICC estimate that is accurate +/±1 to 2 percentage points (depending on the size of the subgroup of patients that the ICC was calculated for). This statement is based on a 95% confidence interval calculated by the method of Zou et. al. [37]. The CoV was defined as the measurement SD divided by the mean caliper (Equation (1)). Similar methods were used to quantify the degree of agreement between two images of the same vessel read by the same reader.

(1) $$\mathrm{CoV}=\frac{\mathrm{SD}}{\mathrm{Mean}}$$

We also examined the mean, SD, CoV, and minimal detectible change (MDC) for vessel caliber measured in repeat pairs of images on the same vessel, and we described the difference in vessel caliber with respect to race, age, glaucoma diagnosis, systemic diabetes mellitus, hypertension, diabetic retinopathy, pseudophakia, and location. SAS 9.4 (Cary, NC, USA) was used for all statistical analysis.

## 3. Results

### 3.1. Demographics

Table 1 illustrates the demographics of the enrolled subjects and image quality distribution. The majority of our sample was females (64%) and African American (68%), and greater than 50 years old (82%). Our sample consisted of eyes that were normal (8 eyes), diagnosed with glaucoma (16 eyes), of which some had advanced primary open-angle glaucoma (POAG) (7 eyes) and mild–moderate POAG (9 eyes), glaucoma suspect (8 eyes), diabetes (4 eyes), diabetic retinopathy with mild–moderate non-proliferate diabetic retinopathy (7 eyes), and hypertensive retinopathy (3 eyes). Six eyes had multiple diagnoses and some patients were monocular. The image quality was roughly equally distributed amongst all three image quality categories with a slight predominance of Fair images.

### 3.2. Agreement in Vessel Caliber Measurements of One Image of the Same Vessel by Two Graders (Inter-Grader)

For the measurements of the vessels around the disc, the mean vessel width for vessels evaluated by both graders, independently, was 81.5 µm with a standard deviation of 33.6 µm. Furthermore, the standard deviation of the variation between graders was estimated to be 10.3 µm. Overall, inter-grader agreement at the disc was excellent at an ICC of 0.90. For measurements of vessels around the macula, the mean vessel width was 37.4 µm with a standard deviation of 9.4 µm. Furthermore, the standard deviation of the variation between graders was estimated to be 5.1 µm. Overall, inter-grader agreement at the macula was good at an ICC of 0.77.

### 3.3. Agreement in Vessel Caliber Measurements of Two Images of the Same Vessel by the Same Grader (Intra-Grader/Inter-Image)

Table 2 illustrates the mean and standard deviation for vessel width measurement of two consecutive images evaluated by the same grader. It also provides the CoV (SD/mean) overall and within subgroups. Vessel calibers for eyes with advanced glaucoma had thinner vessels than mild to moderate or glaucoma suspect. Vessel diameter also became thinner as glaucoma progressed, but with significantly more variability. Vessel calibers of diabetics were smaller than those without. Vessels within the disc were larger than those in the macula. With respect to age, older individuals had smaller vessels. Table 2 also illustrates the degree of agreement between vessel caliber measurements of two consecutive images of the same vessel and within subgroups. ICC was high for all subgroups except for the macula (ICC = 0.72). The CoV of vessel width was also higher in the macula than in the disc. Additionally, the CoV of vessel width was slightly lower in larger (≤52.7 µm) vessels. See Supplemental Materials Tables S1 and S2 for specific values in the macula and optic nerve head region, respectively.

## 4. Discussion

We performed a prospective study evaluating the repeatability of vessel caliber by OCTA. Our study, for the first time, evaluated OCTA repeatability at the level of individual vessels in both the peripapillary and macular retinal vasculature. We found excellent overall inter-session (intra-grader) repeatability of vessel caliber measurements. Our study showed that inter-session inter-image ICC was lower and CoV was higher for the macular retinal vasculature relative to the peripapillary retinal vasculature. There was also a lower CoV for greater vessel width. Taken together, these findings suggest that OCTA may be more reliable for evaluating the larger vessel calibers comprising the optic nerve relative to the smaller vessel calibers comprising the macula.

Our quantitative OCTA analysis showed high repeatability for vessel caliber, having an overall inter-grader agreement of 0.90 and an inter-image ICC of 0.96 for all repeated OCTA images. Previous groups have also evaluated the repeatability of vessel caliber by OCTA. Using an automated approach, Chu et al. demonstrated high repeatability of VDI (coefficient of variance less than 0.031) as measured by spectral-domain OCTA and swept-source OCTA [35]. Kim et al. similarly revealed a high ICC of 0.994 by SD-OCTA based on a semi-automated approach for calculating VDI by SD-OCTA [38]. More recently, Pastore et al., using a similar method, demonstrated good reproducibility and reliability of Heidelberg Spectralis OCTA measurements of VD and foveal avascular zone [39]. Their findings further suggest the utility of OCTA for reliable and reproducible measurements of the retinal vasculature. Although promising, it should be noted that VDI quantifies vessel caliber as an averaged value across the entire image [35]. Therefore, it is possible that localized changes in vessel caliber and of individual vessels may not be sensitively detected by an averaged metric [40,41]. Given these potential shortcomings associated with an averaged metric of an entire scan, our study sought to precisely quantify OCTA vessel caliber repeatability at the level of individual vessels.

The ICC quantifies the measurement variability relative to the variability in vessel caliber between vessels. Though the measurement variability of vessels around the macula and disc was similar in magnitude, there was greater variability in caliber between vessels around the disc. Thus, the measurement variability for the macula vessels constituted a larger proportion of the overall variability, leading to a lower ICC. Our study showed that vessel caliber repeatability was lower for the macular retinal vasculature (ICC of 0.72, CoV of 0.15) relative to the peripapillary retinal vasculature (ICC of 0.96, CoV of 0.07). This is in keeping with prior studies conducted by Eastline et al., which similarly observed lower reproducibility for OCTA metrics including VD and PD for the macular region relative to the peripapillary region [42]. As previously suggested, this lower value may be explained by a higher resolution needed for smaller vessels in the macula considering that the resolution of the Heidelberg Spectralis OCTA is 3.9 µm per pixel [43].

Our study shows thinner vessel width in more severe glaucoma, as well as a higher CoV for vessel width in more severe glaucoma. This is in line with several studies that have also shown an inverse relationship between vessel diameter and glaucoma progression [44,45,46,47]. Our study results also show that diabetics had smaller vessels. However, these findings are contrary to other studies that show a relationship between larger arteriolar vessel calibers and further progression of diabetic retinopathy [48,49,50], indicating that more research needs to be conducted to further explore this relationship. Our study also shows that older individuals had smaller vessels [44,51]. This is consistent with work from Wong et al. showing that vessel caliber decreases by 2 µm for each decade of increase in age [51].

There is less variability in the disc (CoV = 0.07) as compared to the macula (CoV = 0.15) and with larger vessel width, which may be due to the limit in the lateral resolution of the OCT and the difficulty associated with discerning the edges of a given vessel. In our study, vessels in the disc were larger than those in the macula. Although this observation is intuitive, it also highlights that the coefficient of variation is lower in the disc because the mean vessel size is higher.

### Limitations

This study demonstrates good repeatability and reliability of vessel caliber measurements using OCTA. However, there were some limitations of this study that should be addressed. This is a small sample single-center study at an academic institution using a single OCTA device, which may limit its external validity. With a diverse study population of 28 patients and 47 eyes, we were able to subdivide our analysis to look at vessel caliber variables amongst different diseases, demographics, and locations. Given the limited study size, future studies would look further into these subcategories with a larger study population. Our study also demonstrated high inter-grader and intra-grader. However, images were chosen for high quality, vessels were selected for clarity and large in size, and measurements were made carefully using imaging grading software. This leads to possible challenges to the study’s generalizability to clinical practice where motion artifacts and low-quality images are more frequent and adequate time is not available to make careful and precise measurements. Similarly, although the study included diseased eyes, the vessels that were examined were picked for large size and clarity. Thus, the vessels that were measured may not represent the most diseased pathology or those that would be of focus in clinical practice.

OCTA, in its current manifestation, has been primarily used to determine VD and PD metrics for assessing ocular diseases such as glaucoma [34,52,53,54,55,56,57,58,59]. However, these parameters reflect static rather than dynamic changes in blood flow and are generally indicative of permanent, structural changes in the retinal microvasculature. OCTA images are taken over the course of two minutes encapsulating multiple cardiac cycles. This may lead to potential motion artifacts given pulsations in arteries and veins may range from 4 to 10 µm between systole and diastole [20,21,23,24], which represent a 1–2 pixel change in vessel caliber on these OCTA images. This may also explain our finding that vessels of higher caliber have a significantly higher standard deviation of measurement error than smaller vessels. Additionally, vessels are dynamic such that they not only respond to systemic changes such as oxygenation [60], but they also self-regulate vessel caliber and blood flow by way of autoregulation. Nevertheless, vascular flow in smaller capillary vessels has also been suggested to have a significant role in the pathogenesis of ocular diseases [61,62].

The relatively new technology of adaptive optics (AO) provides the ability to study retinal vessel structure with great detail, superior to OCTA. In particular, AO is able to achieve lateral resolutions up to about 2 µm, compared to our OCTA resolution of 3.9 µm [43,63,64]. Prior work has shown that capillaries, as visualized by AO, similarly respond to systemic oxygen challenges with a change in vessel caliber [60,62]. Since VD is a static parameter, these dynamic changes may not be sufficiently captured by OCTA. In contrast, the quality of imaging by OCTA highly depends on the resolution of the machine. The current Heidelberg Spectralis resolution measures 3.6 by 5.7 µm per pixel axially and laterally, respectively [43]. Moreover, since the width of the scanning beam in OCTA cannot be infinitely small, it tends to overestimate caliber measurements by up to two beam widths [33]. Taken together, our finding of a lower ICC for the macula by OCTA suggests that higher-resolution imaging modalities may be necessary for detecting subtle changes in vessel caliber for smaller vessels.

## 5. Conclusions

Overall, OCTA imaging provides reproducible vessel caliber measurements important for determining changes in blood flow, especially in the context of diseases affecting retinal vasculature. Since inter-image ICC was lower in the macula compared to the optic nerve head, OCTA may be more reliable for measuring vessels of larger calibers. Given the dynamic, pulsatile nature of the retinal microvasculature, higher-resolution imaging modalities may be necessary for parsing through the changes in vessel caliber for the smaller vessels comprising the macula.

Seeing that many OCTA-derived measurements are affected by vessel caliber, an improved understanding of vessel caliber repeatability may further enhance the clinical applications of OCTA. Our findings may also be useful for guiding future studies. In particular, a larger sample size is needed to better understand the effects of age, sex, race, diagnosis, image quality, and vessel length on the variability of vessel caliber measurements, as previously suggested [11,65,66].

## Figures and Tables

**Table 1:** Demographic data, diagnoses, and image quality.

Demographics		No. of Patients
**Sex**, n (%)	Female	18 (64%) *
	Male	10 (35%)
**Race**, n (%)	African-American	19 (68%) *
	Caucasian	7 (25%)
	Asian	2 (7%)
**Age**, n (%)	<50 years	5 (18%)
	= or >50 years	23 (82%) *
**Diagnoses**		**No. of eyes**
Glaucoma		16
	Advanced POAG	7
	Mild–moderate POAG	9
Glaucoma Suspect		8
Diabetic Retinopathy	Mild–moderate NPDR	7
Grade I Hypertensive Retinopathy		3
Healthy Controls		8
**Image Quality**		**No. of images**
Poor		52 (31.3%)
Fair		63 (38.0%) *
Good		61 (36.7%)

* Bolded numbers highlight the majority. POAG = primary open-angle glaucoma; NPDR = non-proliferative diabetic retinopathy.

**Table 2:** Mean, SD, ICC, and CoV of Vessel Width Measures of Two Sequential Images of the Same Vessel by the Same Grader.

	Mean Vessel Width for Each Subgroup (μm)	Standard Deviation of Two Images ^1^ (μm)	CoV of Vessel Width	ICC ˆ (95% Confidence Interval)	MDC
All Subjects (n = 550)	54.48	5.28	0.10	0.96	14.08
Age *					
<55 (n = 215)	59.39	6.01	0.10	0.95 (0.95, 0.96)	16.04
55+ (n = 335)	51.32	4.75	0.09	0.97 (0.96, 0.97)	12.67
Race					
White (n = 155)	60.03	3.35	0.06	0.98 (0.98, 0.98)	8.89
Asian (n = 40)	55.31	6.62	0.12	0.89 (0.88, 0.91)	17.43
Black (n = 355)	51.96	5.77	0.11	0.96 (0.95, 0.96)	15.44
Sex					
Female (n = 335)	55.75	4.54	0.08	0.97 (0.97, 0.98)	12.10
Male (n = 215)	52.49	6.26	0.12	0.94 (0.94, 0.95)	16.67
Glaucoma *					
None (n = 295)	54.14	5.63	0.10	0.95 (0.95, 0.96)	14.99
Suspect (n = 115)	61.77	4.35	0.07	0.96 (0.95, 0.97)	11.50
Mild/mod (n = 85)	51.16	4.91	0.10	0.97 (0.97, 0.98)	13.13
Advanced (n = 55)	46.15	5.63	0.12	0.96 (0.96, 0.97)	15.05
Diabetes *					
0 (n = 340)	57.44	4.93	0.10	0.96 (0.96, 0.97)	13.11
1 (n = 210)	49.68	5.80	0.12	0.95 (0.94, 0.96)	15.46
Hypertension					
0 (n = 205)	57.68	5.58	0.10	0.95 (0.94, 0.96)	14.85
1 (n = 345)	52.57	5.09	0.10	0.96 (0.96, 0.97)	13.55
Pseudophakia					
0 (n = 480)	55.01	5.31	0.10	0.96 (0.95, 0.97)	14.16
1 (n = 70)	50.80	5.08	0.10	0.96 (0.95,0.96)	13.53
Vessel Width *					
Low (<33.4) (n = 183)	28.29	3.18	0.11		
Medium (33.4 = 52.7) (n = 184)	40.97	4.21	0.10		
High (52.7+) (n = 183)	94.23	7.47	0.08		
Length †					
Less than median (n = 281)	55.24	4.88	0.09	0.97 (0.96, 0.97)	13.05
More than median(n = 269)	53.68	5.67	0.11	0.95 (0.94, 0.96)	15.10
Location *					
Disc (n = 240)	80.29	5.58	0.07	0.98 (0.98, 0.98)	15.07
Macula (n = 210)	34.49	5.03	0.15	0.72 (0.67, 0.76)	12.48

* Statistically significant difference in mean vessel width to the level of *p* < 0.05. † Based on the lowest quality of the two images. **ˆ** ICC estimates are accurate +/±1 to 2 percentage points based on the method of Zou [37]. ^1^SD of two images refers to the SD of measurements of two images on the same vessel by the same grader; ICC—interclass correlation coefficient; CoV—coefficient of variation; MDC—Minimal Detectible Change.

## Data Availability

The data presented in this study are available on request from the corresponding author. The data are not publicly available due to patient privacy.

## Supplementary Materials

The following supporting information can be downloaded at: https://www.mdpi.com/article/10.3390/vision7030048/s1, Figure S1: Example of Good Quality Image; Figure S2: Example of Fair Quality Image; Figure S3: Example of Poor Quality Image; Table S1: Mean, SD, ICC, and CoV of Vessel Width Measures of Two Sequential Images of the Same Vessel in the Macula by the Same Grader; Table S2: Mean, SD, ICC, and CoV of Vessel Width Measures of Two Sequential Images of the Same Vessel at the Optic Nerve Head by the Same Grader.
