# Supplementary material for: Repeatability and Comparability of Retinal Blood Vessel Caliber Measurements by OCTA
Source: Vision (Basel). 2023 Jul 3;7(3):48. doi: 10.3390/vision7030048 (PMC10366731; doi:10.3390/vision7030048)
Supplement: Supplementary file 1 [file vision-07-00048-s001.zip › vision-1903378-supplementary.pdf]

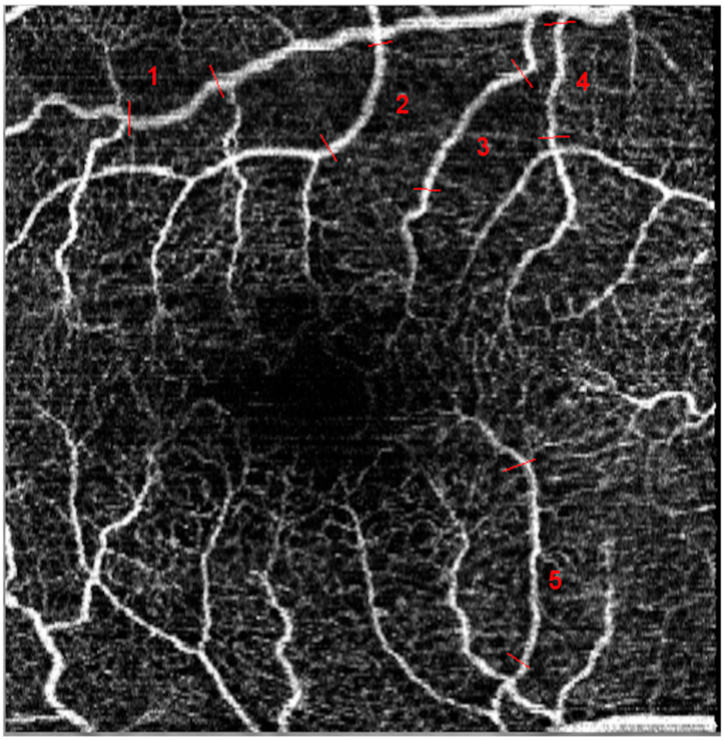

Figure S1. - Example of Good Quality Image.

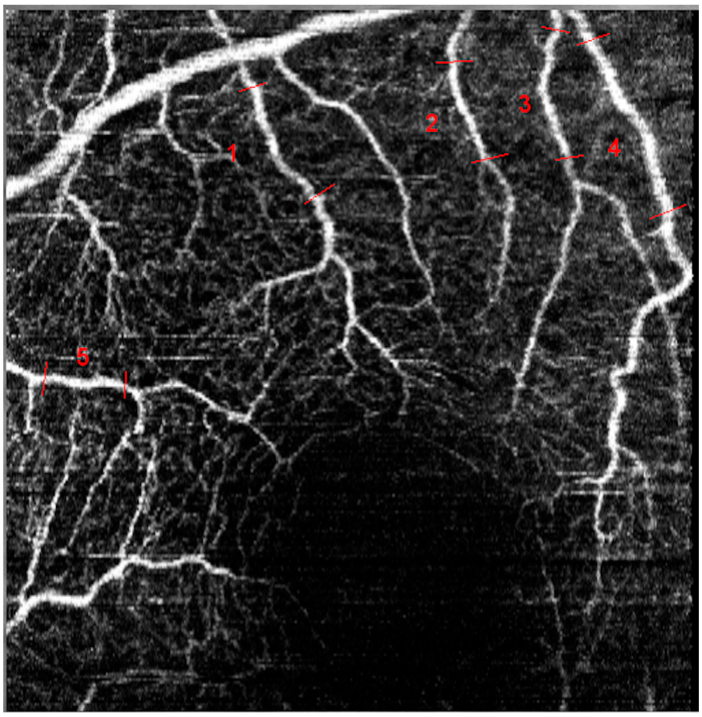

Figure S2. - Example of Fair Quality Image.

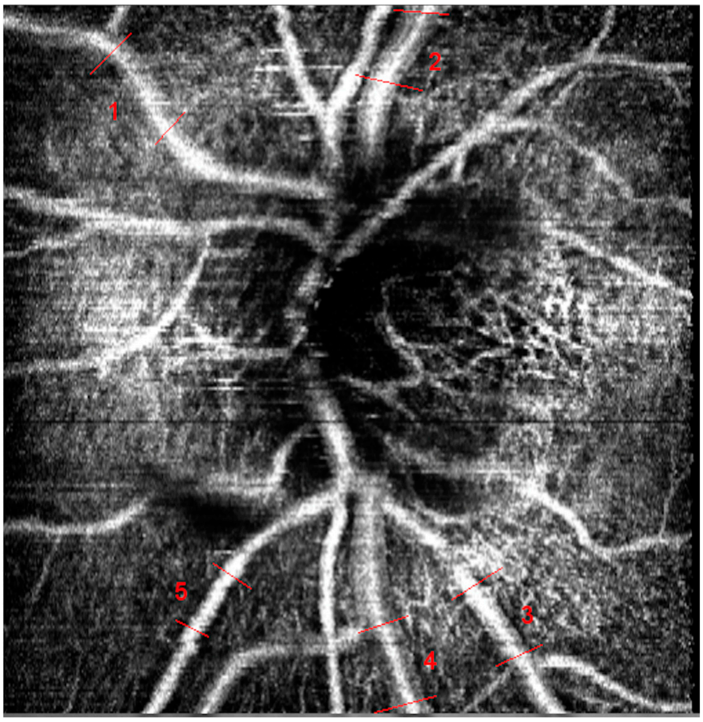

Figure S3. - Example of Poor Quality Image.

Table S1. Mean, SD, ICC, and CoV of Vessel Width Measures of Two Sequential Images of the Same Vessel in the Macula by the Same Grader.

	Mean Vessel Width for each subgroup (µm)	Standard Deviation of two images¹ (µm)	CoV of Vessel Width	ICC^ (95% Confidence Interval)	MDC
All Subjects (n=310)	34.49	5.03	0.15	0.72 (0.67, 0.76)	13.95
Age*					
<55 (n=115)	36.40	6.65	0.18	0.62 (0.57, 0.67)	18.43
55+ (n=195)	33.37	3.77	0.11	0.80 (0.77, 0.83)	10.44
Race*					
White (n=80)	34.22	2.48	0.07	0.87 (0.85, 0.89)	15.17
Asian (n=20)	42.06	5.47	0.13	0.84 (0.81, 0.86)	15.72
Black (n=210)	33.88	5.67	0.17	0.64 (0.60, 0.69)	6.89
Sex					
Female (n=185)	24.03	3.62	0.11	0.81 (0.78, 0.84)	10.03
Male (n=125)	35.17	6.59	0.19	0.63 (0.58, 0.68)	18.26
Glaucoma*					
None (n=165)	34.06	5.78	0.17	0.67 (0.62, 0.71)	16.02
Suspect (n=60)	36.22	2.97	0.08	0.81 (0.78, 0.84)	8.24
Mild/mod (n=45)	31.85	2.80	0.09	0.89 (0.88, 0.91)	7.76
Advanced (n=40)	36.64	6.04	0.16	0.68 (0.64, 0.73)	16.74
Diabetes					
0 (n=190)	33.80	5.23	0.15	0.63 (0.58, 0.68)	14.48
1 (n=120)	35.59	4.71	0.13	0.80 (0.77, 0.83)	13.06
Hypertension					
0 (n=120)	34.75	6.28	0.18	0.57 (0.51, 0.63)	17.41
1 (n=190)	34.44	4.05	0.12	0.81 (0.78, 0.84)	11.23
Pseudophakia					
0 (n=265)	24.62	5.11	0.15	0.72 (0.68, 0.76)	14.16

1 (n=45)	33.74	4.57	0.14	0.63 (0.58, 0.69)	12.66
Vessel Width*					
Low (<33.4) (n=166)	28.32	3.25	0.11	#	9.01
Medium (33.4=52.7) (n=132)	39.88	3.91	0.10		10.85
High (52.7+) (n=12)	60.58	18.43	0.30		51.09
Length *					
Less than median (n=155)	32.67	3.91	0.12	0.78 (0.75, 0.82)	10.84
More than median(n=155)	36.31	5.95	0.16	0.65 (0.60, 0.70)	16.49

*Statistically significant difference in mean vessel width to the level of $P < 0.05$. ^ICC confidence intervals calculated using the method of Zou [37]. #ICC for vessel width categories not provided because stratifying by vessel width artificially reduces the between-vessel variance on which the ICC depends. SD of two images refers to the SD of measurements of two images on the same vessel by the same grader.; ICC-interclass correlation coefficient; CoV-coefficient of variation; MDC- Minimal Detectible Change.

Table 2. Mean, SD, ICC, and CoV of Vessel Width Measures of Two Sequential Images of the Same Vessel at the Optic Nerve Head by the Same Grader.

	Mean Vessel Width for each subgroup (μm)	Standard Deviation of two images[1] (μm)	CoV of Vessel Width	ICC^ (95% Confidence Interval)	MDC
All Subjects (n=240)	80.29	5.58	0.07	0.98 (0.98, 0.98)	15.47
Age					
<55 (n=100)	85.83	5.18	0.06	0.98 (0.98, 0.98)	14.36
55+ (n=140)	76.33	5.84	0.08	0.98 (0.98, 0.98)	16.21
Race*					
White (n=75)	87.56	4.08	0.05	0.99 (0.98, 0.99)	21.07
Asian (n=20)	68.56	7.60	0.11	0.91 (0.90, 0.92)	16.38
Black (n=145)	78.15	5.91	0.08	0.98 (0.98, 0.98)	11.31
Sex					
Female (n=150)	82.53	5.46	0.07	0.98 (0.98, 0.98)	15.14
Male (n=90)	76.56	5.77	0.08	0.98 (0.97, 0.98)	16.00
Glaucoma*					
None (n=130)	79.62	5.44	0.07	0.98 (0.98, 0.98)	15.08
Suspect (n=55)	89.65	5.46	0.06	0.97 (0.96, 0.97)	15.14
Mild/mod (n=40)	72.89	6.51	0.09	0.98 (0.97, 0.98)	18.06
Advanced (n=15)	71.52	4.34	0.06	0.99 (0.99, 0.99)	12.02
Diabetes*					
0 (n=150)	87.39	4.52	0.05	0.99 (0.98, 0.99)	12.53
1 (n=90)	68.46	7.00	0.10	0.96 (0.96, 0.97)	19.40
Hypertension*					
0 (n=85)	90.04	4.39	0.05	0.99 (0.98, 0.99)	12.18
1 (n=155)	74.94	6.13	0.08	0.98 (0.97, 0.98)	17.00
Pseudophakia					
0 (n=215)	80.15	5.54	0.07	0.98 (0.98, 0.98)	15.36
1 (n=25)	81.51	5.90	0.07	0.98 (0.97, 0.98)	16.34
Vessel Width*					
Low (<33.4) (n=17)	28.05	2.41	0.09		6.69
Medium (33.4-52.7) (n=52)	43.75	4.89	0.11	#	13.57
High (52.7+) (n=171)	96.59	5.99	0.06		16.59
Length *					
Less than median (n=126)	83.00	5.86	0.07	0.98 (0.97, 0.98)	16.23
More than median(n=114)	77.29	5.26	0.07	0.98 (0.98, 0.98)	14.57

*Statistically significant difference in mean vessel width to the level of $P < 0.05$. ^ICC confidence intervals calculated using the method of Zou [37]. #ICC for vessel width categories not provided because stratifying by vessel width artificially reduces the between-vessel variance on which the ICC depends. SD of two images refers to the SD of measurements of two images on the same vessel by the same grader.; ICC-interclass correlation coefficient; CoV-coefficient of variation; MDC- Minimal Detectible Change.
